# Disaster Management in Hemodialysis Centers: Ensuring Continuity of Lifesaving Care

**DOI:** 10.1055/s-0046-1827454

**Published:** 2026-08-20

**Authors:** Muhammad N. Hashmi, Saad Almarzouk, Fayez Hejaili, Hammad Raza

**Affiliations:** 1 King Abdullah Dialysis Centre, Ministry of National Guard Health Affairs, Jeddah, Saudi Arabia; 2 King Abdullah International Medical Research Centre (KAIMRC), Riyadh, Saudi Arabia; 3 King Abdulaziz Medical City, King Abdullah International Medical Research Centre (KAIMRC), Riyadh, Saudi Arabia

**Keywords:** hemodialysis, disaster preparedness, emergency response, health system resilience, continuity of care, water supply, power outage

## Abstract

Hemodialysis is a life-sustaining therapy that depends on uninterrupted access to electricity, clean water, medical supplies, and trained staff. This dependence makes hemodialysis units uniquely vulnerable to disasters such as natural catastrophes, power failures, pandemics, or civil emergencies. The COVID-19 pandemic, major earthquakes, and large-scale floods have highlighted the fragility of dialysis infrastructure worldwide. This review synthesizes available evidence on disaster preparedness, response, and recovery in hemodialysis centers. Drawing on international experiences and existing guidelines, it proposes a framework to enhance resilience and ensure continuity of dialysis care during crises. Key strategies include structured risk assessment, patient and staff education, backup systems for power and water, coordinated regional response networks, and incorporation of disaster readiness into routine dialysis operations. Strengthening preparedness at every level—from the individual center to national health systems—is vital to prevent avoidable morbidity and mortality among this highly vulnerable population.

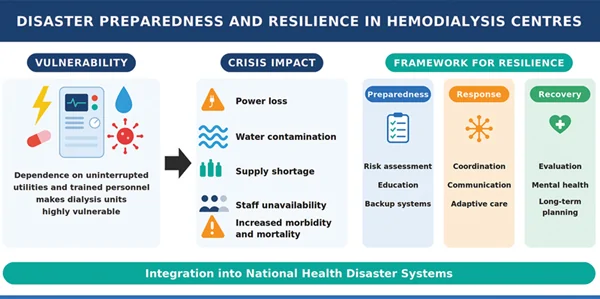

## Introduction

Hemodialysis (HD) represents one of the most technologically dependent forms of chronic therapy. Patients with end-stage kidney disease (ESKD) require regular, uninterrupted access to this treatment—typically three sessions per week. Each treatment depends on functional machines, sterile supplies, pure water, reliable electricity, and trained staff. Any interruption, even for a few days, can result in life-threatening complications such as hyperkalemia, pulmonary edema, or uremia.

Disasters—whether natural, technical, or biological—can instantly disrupt this delicate balance. The global dialysis community has repeatedly faced such challenges: from the massive infrastructure losses during Hurricane Katrina (2005) 1 to the Fukushima earthquake (2011) 2 and the recent COVID-19 pandemic, 3 which redefined infection control and workforce resilience. These events demonstrated that dialysis continuity is not merely a clinical concern but a systemic emergency preparedness issue.

The disruption of infrastructure, including power, water supply, transportation, and communication, frequently leads to the closure or reduced functionality of dialysis centers, resulting in missed or delayed dialysis sessions and increased morbidity and mortality among patients dependent on maintenance HD. 4 5 6 7 8 9 Direct consequences include an increased risk of life-threatening complications such as hyperkalemia, volume overload, and uremia due to missed treatments, as well as a surge in acute kidney injury (AKI) cases, particularly following disasters associated with crush syndrome (e.g., earthquakes). 5 9 In the aftermath of such events, dialysis centers often face shortages of equipment, supplies, and experienced personnel, further compromising care delivery. 9 Patients with comorbidities such as diabetes, cardiovascular, or cerebrovascular disease are at particularly elevated risk for adverse outcomes and mortality in disaster settings. 8 10

Indirect effects encompass barriers to healthcare access, including affordability, displacement, and travel limitations, which are exacerbated in conflict zones and severely affected rural areas. 4 6 10 Disasters also precipitate significant psychological distress among dialysis patients, with high rates of anxiety, depression, and posttraumatic stress disorder reported. 4 7 Pediatric and transplant populations face unique vulnerabilities, including medication shortages and increased infection risk. 5 11

Lessons learned from disasters for HD include the need for individual patient preparedness (e.g., packing an emergency bag), health system readiness (e.g., emergency generators, communication plans, backup supplies), and staff training on procedures like patient evacuation and emergency dialysis. Disasters disrupt dialysis care through power outages and lack of clean water, and can increase hospitalizations and posttraumatic stress for patients, making comprehensive planning crucial at all levels.

Despite repeated lessons, many dialysis facilities still lack comprehensive disaster plans. This review aims to synthesize existing knowledge, highlight best practices, and outline a structured approach to disaster management in HD centers.

### Methodology

This study was conducted as a structured narrative review following the principles of the Scale for the Assessment of Narrative Review Articles (SANRA). The objective was to synthesize current evidence regarding disaster preparedness, emergency response, and recovery strategies in HD centers, with a focus on maintaining continuity of care during natural disasters, pandemics, infrastructure failures, and armed conflicts.

A comprehensive literature search was performed in PubMed/MEDLINE, Scopus, and Google Scholar for studies published between January 2000 and December 2025. Additional references were identified through manual screening of bibliographies from relevant articles and guideline documents.

Search terms included combinations of keywords and Medical Subject Headings (MeSH) such as:

**Table:** 

“hemodialysis”	“hurricane”
“dialysis”	“heat wave”
“end-stage kidney disease”	“pandemic”
“renal replacement therapy”	“COVID-19”
“disaster preparedness”	“armed conflict”
“emergency management”	“war”
“earthquake”	“health system resilience”
“flood”	“continuity of care”

Articles were included if they:

- Reported the impact of disasters or emergencies on HD services or dialysis-dependent patients.
- Described preparedness, mitigation, response, or recovery strategies relevant to dialysis care.
- Were original research articles, systematic reviews, narrative reviews, consensus statements, clinical guidelines, or reports from recognized health organizations.
- Were published in English.
- Studies focusing exclusively on non-dialysis populations or lacking relevance to disaster management in kidney care were excluded.

Titles and abstracts retrieved from the search were screened for relevance. Full-text articles meeting the inclusion criteria were reviewed in detail. Preference was given to high-quality evidence, recent publications, international guidelines, and studies reporting real-world experiences from major disaster events.

Relevant information was extracted regarding:

- Type of disaster or emergency.
- Geographic location and healthcare setting.
- Impact on dialysis infrastructure and service delivery.
- Clinical outcomes among dialysis patients.
- Preparedness and mitigation strategies.
- Response and recovery interventions.

Due to heterogeneity in study designs, disaster types, and reported outcomes, a quantitative meta-analysis was not feasible. Therefore, findings were synthesized narratively and organized into thematic categories, including disaster-specific impacts, preparedness frameworks, emergency response strategies, recovery measures, technological innovations, and ethical considerations.

## Types of Disasters and Their Impact on Hemodialysis Services

**Fig. 1:**
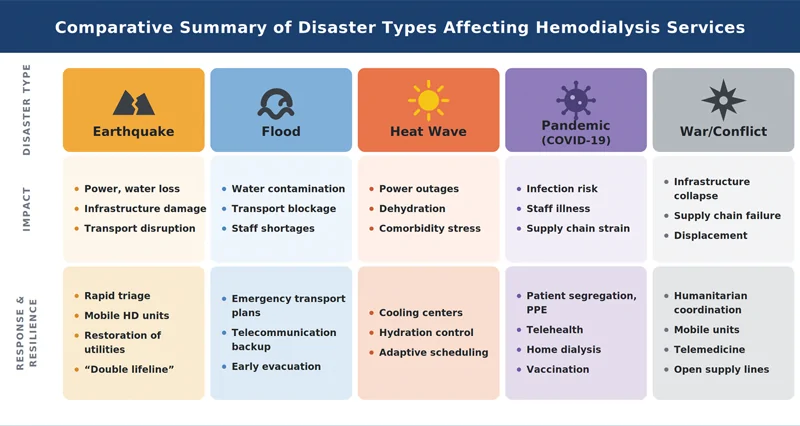
Summary of disasters affecting hemodialysis service.

Hemodialysis units are vulnerable to a broad spectrum of disasters that can compromise infrastructure, supply chains, or personnel. Fig. 1 summarizes the most common threats and their consequences.

### Earthquake

Earthquakes significantly impact HD services, affecting patients with end-stage renal disease (ESRD) and AKI, especially from crush syndrome. The primary challenges arise from infrastructure damage, including loss of power and water supply, destruction of dialysis facilities, and disruption of transportation networks, all of which can severely curtail the ability to deliver HD. 5 9 For example, after the Marmara earthquake, the number of HD sessions in affected centers dropped by nearly 50% in the first week, with a gradual recovery over subsequent months as infrastructure was restored and patients were relocated or transferred to functioning units. 12 Patients with crush-related AKI present a surge in demand for HD, often with more frequent and complicated sessions due to severe metabolic derangements such as hyperkalemia, rhabdomyolysis, and volume overload. These patients are at higher risk for HD complications, including clotting of dialyzer membranes and intradialytic hypotension, necessitating heightened vigilance and resource allocation. 9 13 14 Hyperkalemia is the most common initial indication for HD in this population. 9

Strategies to mitigate this include reducing session frequency, strict dietary and fluid restrictions, and preemptive administration of potassium binders or extra sessions. 12 14 Patient education and disaster preparedness, including emergency kits and clear communication protocols, are essential to optimize compliance and outcomes. 5 14 Restoration of water and power supplies to dialysis units is a top priority, as HD is highly dependent on these utilities. Some centers have implemented double-lifeline systems, such as emergency generators and well water supplies, to enhance resilience. 15 Coordination among regional and national authorities, as well as flexible use of peritoneal dialysis (which is less dependent on infrastructure but has its own challenges), can further support continuity of renal replacement therapy. 5 16

### Floods

Floods have a substantial impact on HD services, primarily through disruption of critical infrastructure, including transportation, water supply, electricity, and communication networks. These disruptions can lead to missed dialysis sessions, increased emergency department visits, higher hospitalization rates, and elevated short-term mortality among patients with ESRD who depend on regular HD.

Floods often damage roads and public transport, impeding patient access to dialysis centers and complicating the delivery of supplies and staff mobilization. 6 16 Water and power outages directly affect the ability of dialysis units to operate, as both are essential for the safe provision of HD. 6 16 Communication breakdowns further hinder coordination between facilities, emergency services, and patients. 6

Flood-related disasters often lead to missed dialysis sessions, with up to 44% of patients missing at least one session. Patients with less experience, living alone, or unaware of emergency plans are at higher risk. 17 18 Floods exacerbate vulnerabilities in the dialysis population, particularly those with comorbidities, and displacement from family and social supports contributes to adverse outcomes.

Effective disaster preparedness—including patient education, facility emergency planning, inter-institutional collaboration, and telemedicine deployment—can reduce the incidence of missed dialysis and adverse outcomes. 6 16 18 However, literature consistently highlights the fragility of dialysis service continuity during floods and the need for tailored, region-specific disaster planning integrated into broader public health frameworks. 6 16

### Heat Waves

Heat waves have a significant impact on patients receiving HD, primarily through increased morbidity and mortality. Large-scale epidemiological studies demonstrate that exposure to extreme heat events is associated with a higher risk of hospital admission and death among patients with ESRD undergoing maintenance dialysis. For example, a multicenter study in major U.S. cities found that extreme heat events increased the rate of same-day hospital admission (rate ratio [RR]: 1.27, 95% CI: 1.13–1.43) and same-day mortality (RR: 1.31, 95% CI: 1.01–1.70) in this population, with particularly elevated risk among those with comorbidities such as congestive heart failure, chronic obstructive pulmonary disease, or diabetes. 19 These risks may vary by geographic region and race/ethnicity, suggesting that local climate and sociodemographic factors modulate vulnerability.

A recent national cohort study further corroborates these findings, showing that during periods of extreme humid-heat (heat index >40.6°C for ≥2 days or >46.1°C for ≥1 day), patients receiving dialysis in urban areas experienced an 18% increase in mortality risk (hazard ratio: 1.18, 95% CI: 1.15–1.20). The effect was most pronounced in the Southeast United States, highlighting regional differences in susceptibility. 20

While heat waves are associated with increased renal morbidity and AKI in the general population, evidence from Australia indicates that hospitalizations for dialysis specifically do not necessarily increase during heat waves, suggesting that the primary impact is on acute complications and comorbid conditions rather than on the direct utilization of dialysis services. 21 However, the overall burden of kidney-related morbidity and mortality rises with increasing temperature, and the risk escalates with the intensity of heat waves. 22

Operationally, these findings imply that HD services must anticipate increased acute care needs during heat waves, particularly for patients with multiple comorbidities and in regions with higher heat exposure. Preventive strategies—including patient education on fluid management, close monitoring for heat-related complications, and contingency planning for service disruptions—are warranted to mitigate these risks. The literature underscores the importance of targeted interventions and resource allocation to protect this highly vulnerable population during periods of extreme heat. 19 20 22

### COVID-19 Impact

COVID-19 has had a profound impact on HD services, affecting both clinical outcomes and the organization of care. Patients receiving in-center HD are at increased risk for SARS-CoV-2 infection due to their inability to distance physically and the necessity of frequent visits to healthcare facilities, which has resulted in higher rates of infection, hospitalization, and mortality compared to those on home dialysis modalities. 23 24 25 The risk is further amplified for patients residing in skilled nursing facilities, where the odds of COVID-19-related adverse events were markedly higher, especially early in the pandemic. 25

The pandemic led to significant operational challenges for dialysis centers, including the need for rapid implementation of infection control measures such as routine screening, patient segregation, and universal masking for staff. 23 These measures were effective in reducing transmission within dialysis units but created substantial service pressures, including increased demand for isolated dialysis sessions and inpatient beds, as well as high rates of staff illness. 23 26 Simulation modeling has demonstrated that maintaining outpatient capacity and rapidly reallocating patients are critical to managing surges in COVID-19-positive cases, with patient transport logistics also emerging as a major bottleneck. 26

There was a notable decline in the incidence of patients initiating dialysis and kidney transplantation during the early phase of the pandemic, reflecting disruptions in access to care and changes in clinical decision-making. Patients tended to start dialysis at lower eGFRs and were more likely to initiate therapy with central venous catheters, indicating delayed or suboptimal initiation of renal replacement therapy. 27 Psychological distress among dialysis patients increased, with preventive measures such as isolation and reduced face-to-face contact contributing to anxiety, sadness, and loneliness, particularly among those on home modalities. The introduction of telemedicine and changes in clinical routines created communication barriers and reduced direct physical contact between patients and providers, highlighting the need for more frequent psychological evaluation and proactive discussion about modality selection. 28

In summary, COVID-19 has exposed vulnerabilities in HD service delivery; necessitated rapid adaptation of infection control protocols; and underscored the importance of modality selection, psychological support, and vaccination strategies for this high-risk population.

### War Impact

Armed conflict has a profound and multifaceted impact on HD services, resulting in increased morbidity and mortality among patients with ESKD who depend on regular dialysis for survival. The disruption of healthcare infrastructure, damage to dialysis facilities, interruption of supply chains for consumables and medications, and displacement of both patients and healthcare workers are consistently reported consequences in conflict zones such as Sudan, Gaza, Syria, and other regions.

A substantial proportion of patients are unable to maintain their regular dialysis schedules due to facility closures, equipment damage, power outages, and staff shortages. For example, in Sudan and Gaza, more than half of patients missed scheduled sessions, with interruptions sometimes lasting over a week, leading to acute complications and increased hospitalization rates. 4 29 30 Missed or reduced dialysis sessions result in a high incidence of complications such as intradialytic hypotension, volume overload, electrolyte disturbances, and infections. Mortality rates among dialysis-dependent patients in besieged or severely affected regions can approach or exceed 50%, as documented in multiple conflict settings. 30 31

Conflicts disrupt the supply of essential consumables (dialyzers, fluids), medications (antihypertensives, insulin), and even basic utilities (electricity, water), directly impacting the ability to deliver safe and effective dialysis. 30 31 32 Displacement and system breakdowns often result in loss of prior medical records and reduced access to nephrologists, further complicating care and patient education. 30 33

Outcomes vary depending on the degree of infrastructure resilience and external humanitarian support. Regions with sustained international aid and intact logistics (e.g., parts of Ukraine) have demonstrated the ability to maintain and even expand dialysis services during conflict, underscoring the importance of preparedness, coordination, and open supply lines. Telemedicine and patient education are proposed as adjuncts to mitigate some challenges. 4 30 31 32 Table 1 summarizes impact of different disasters on dialysis patients.

**Table 1:** Impact of different disasters on patients' dialysis

Disaster type	Key quantitative findings
Earthquake	∼50% reduction in HD sessions during the first week after the Marmara earthquake
Floods	Up to 44% of patients missed at least one dialysis session
Heat waves	27% increase in same-day hospital admissions; 31% increase in mortality risk
Extreme humid heat	HR for mortality 1.18 (95% CI: 1.15–1.20)
COVID-19	Higher hospitalization and mortality in in-center HD compared with home dialysis
War zones	>50% of patients missed dialysis sessions; mortality approaching 50% in severely affected regions

## Framework for Disaster Management in Hemodialysis Centers

Disaster management in HD centers is structured around a comprehensive framework that addresses preparedness, response, and recovery phases, with the goal of maintaining continuity of care for patients with ESKD and those with AKI who require renal replacement therapy. The framework is informed by lessons from natural disasters, pandemics, and mass casualty events, and is supported by consensus recommendations and real-world experiences. 5 14 34 35

Key components of the disaster management framework include the following:

1. **Pre-disaster preparedness** :
▪ *Facility-level planning* : Dialysis centers must maintain and regularly update disaster plans, which include identification of partner facilities for patient transfer, robust communication strategies that account for potential loss of telecommunication infrastructure, and protocols for staff redistribution in case of shortages. 14 34 35
▪ *Patient education and empowerment* : Patients should be educated about emergency diets (e.g., low potassium, low fluid intake), evacuation procedures, and the importance of carrying medical documents and medications. Early evacuation planning is critical for predictable disasters (e.g., hurricanes). 14 34
▪ *Resource stockpiling and infrastructure resilience* : Centers should ensure backup supplies of consumables, access to alternative water and power sources, and emergency kits for patients. Mobile dialysis units and interoperable patient registries enhance system resilience. 5 35
▪ *Special populations* : Pediatric patients, transplant recipients, and displaced persons require tailored protocols due to unique vulnerabilities. 5 11
2. **Disaster response** :
▪ *Coordination and communication* : Real-time coordination among dialysis facilities, regional networks, and emergency response agencies is essential. Daily conference calls and centralized information sharing facilitate resource allocation and triage. 34 35
▪ *Triage and allocation* : In resource-limited scenarios, ethical frameworks guide allocation based on urgency and prognosis, balancing the needs of acute and maintenance dialysis patients. Algorithms for triage may be employed to optimize outcomes. 36
▪ *Adaptation of dialysis prescriptions* : When access is compromised, the frequency and duration of dialysis sessions may be reduced temporarily, and potassium binders may be used as adjuncts. Patients may be redirected to alternative modalities (e.g., peritoneal dialysis) if feasible. 5 14
▪ *Infrastructure restoration* : Priority is given to restoring water and power to dialysis units, as interruption of these services is life-threatening for patients unable to evacuate. 14
3. **Post-disaster recovery** :
▪ *Patient tracking and continuity of care* : Interoperable registries and coordinated follow-up are necessary to track displaced patients and ensure ongoing care. 5 35
▪ *Addressing physical and mental health needs* : Post-disaster interventions should address neglected medical issues, psychological distress, and social challenges, especially in pediatric and displaced populations. 11
▪ *System evaluation and improvement* : After-action reviews inform updates to disaster plans and preparedness protocols. 34 35

**Fig. 2:**
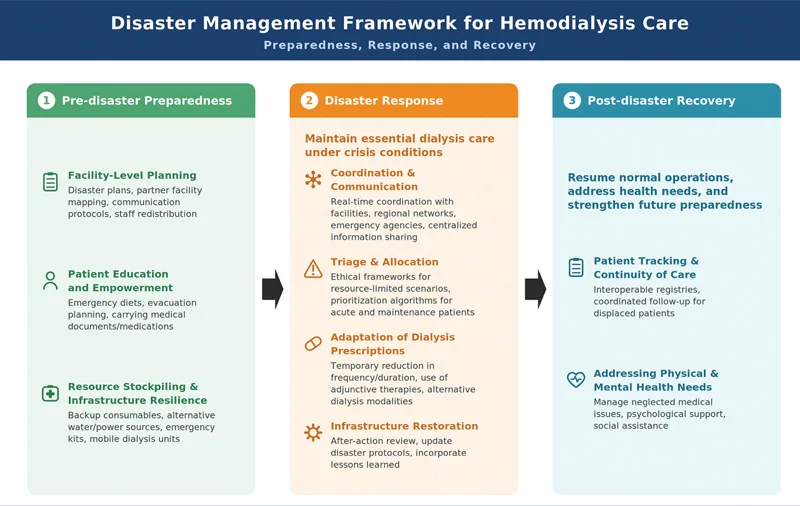
Disaster management framework.

This framework is dynamic and must be adapted to the specific context of the disaster, local resources, and patient population. Integration of nephrology services into broader disaster management systems is recognized as both a clinical and humanitarian imperative. 5 35 Fig. 2 represents the framework.

## Technological and Policy Innovations

Disaster management in HD has seen significant technological and policy innovations aimed at ensuring continuity of care for patients with ESRD during crises such as natural disasters, pandemics, or infrastructure failures. Water conservation and management have become central to disaster preparedness in dialysis. Innovations in circular water management—encompassing reduction, reuse, and recycling of water—are being advocated to mitigate the vulnerability of HD to water shortages. Strategies include optimizing reverse osmosis performance, reducing dialysate flow rates, and repurposing reject water for potable or agricultural use. These approaches are supported by policy recommendations that emphasize sustainability and resilience in dialysis infrastructure, with the goal of integrating water-saving practices into routine and emergency protocols. 37

Remote and virtual technologies have also transformed disaster response for home dialysis patients. Telehealth and remote patient monitoring, accelerated by regulatory changes and the COVID-19 pandemic, enable ongoing clinical oversight and support, even when in-person visits are not feasible. The integration of artificial intelligence (AI) and advanced data analytics into remote monitoring platforms is anticipated to further personalize and optimize dialysis care during emergencies. 38 Device innovation in home HD includes machines with on-demand dialysate generation, automated safety features, and remote monitoring capabilities. These advances facilitate decentralized dialysis delivery, reducing dependence on centralized facilities and infrastructure, and improving access during disasters. The development of wearable and portable dialysis devices is ongoing, with the potential to further enhance mobility and flexibility for patients in crisis situations. 39

In summary, disaster management in HD is being shaped by innovations in alternative dialysis modalities (such as peritoneal dialysis), water conservation strategies, remote monitoring and telehealth, advanced home dialysis devices, and supportive policy changes. These developments collectively enhance the ability to maintain dialysis care during emergencies and mitigate the impact of infrastructure disruptions on vulnerable patient populations.

## Role of Government and Health Systems in Disaster Preparedness

Effective disaster preparedness for HD services extends beyond individual dialysis facilities and requires coordinated action at governmental and health-system levels. National and regional health authorities play a central role in ensuring continuity of renal replacement therapy during emergencies through policy development, infrastructure investment, workforce planning, and emergency response coordination.

Governments should integrate dialysis services into national disaster preparedness and emergency response frameworks. Key responsibilities include the following:

- Establishing national policies and standards for disaster preparedness in dialysis facilities.
- Developing regional networks of dialysis centers to facilitate patient transfer and resource sharing during emergencies.
- Maintaining strategic reserves of essential supplies, including dialyzers, dialysis fluids, vascular access materials, medications, and water-treatment equipment.
- Prioritizing dialysis facilities for restoration of electricity, water supply, fuel, transportation, and telecommunications following disasters.
- Supporting interoperable electronic health records and patient registries to facilitate continuity of care for displaced patients.
- Providing financial mechanisms and emergency funding to support disaster response and facility recovery.
- Government agencies should also collaborate with disaster management authorities, humanitarian organizations, and professional nephrology societies to ensure coordinated responses during large-scale emergencies.

## Ethical and Clinical Considerations

During severe disasters, limited resources may force difficult triage decisions. Ethical frameworks must guide rationing while upholding fairness and transparency. Communication with patients and families is crucial to maintain trust. Protection of vulnerable populations—such as elderly, disabled, or low-income patients—must remain a moral priority.

### Challenges in Low-Resource Countries

The implementation of disaster preparedness measures remains particularly challenging in low- and middle-income countries (LMICs), where healthcare systems often operate under significant resource constraints. Common challenges include the following:

- Limited numbers of dialysis centers and nephrology specialists.
- Chronic shortages of dialysis consumables and medications.
- Unreliable electricity and water infrastructure.
- Weak transportation and communication networks.
- Inadequate health information systems and patient registries.
- Insufficient funding for emergency preparedness activities.
- Dependence on imported dialysis supplies vulnerable to supply-chain disruptions.
- Political instability, armed conflict, and humanitarian crises.
- During disasters, these vulnerabilities may result in prolonged interruption of dialysis services, increased hospitalization rates, and preventable mortality among patients with ESKD.

### Strategies for Resource-Limited Settings

To improve resilience in low-resource settings, governments and healthcare organizations should prioritize cost-effective interventions, including the following:

- Development of regional emergency dialysis networks.
- Establishment of minimum emergency stockpiles of dialysis supplies.
- Use of mobile dialysis units where feasible.
- Community-based patient education programs focused on emergency preparedness.
- Strengthening local manufacturing and procurement systems for essential consumables.
- Adoption of telehealth solutions for patient monitoring and coordination.
- Partnerships with international organizations and humanitarian agencies to support surge capacity during major disasters.

Investment in health-system resilience should be viewed not only as emergency preparedness but also as a critical component of universal health coverage and equitable access to life-sustaining renal care.

## Future Directions

▪ AI-driven models have demonstrated substantial utility in predicting acute risks and adverse events among HD patients, which is directly relevant to disaster preparedness and proactive patient transfer or evacuation planning. Several machine learning approaches have been validated for real-time and near-real-time risk stratification using routinely available clinical, physiological, and device-derived data.
▪ Conduct **multicenter research** on the outcomes of disaster preparedness interventions.
▪ Promote **international collaboration** under global kidney alliances for resource mobilization and knowledge sharing.
▪ Development of redundant power and water systems.
▪ Establishment of regional referral and mutual-aid networks.
▪ Maintenance of interoperable electronic patient registries.
▪ Diversification of dialysis modalities, including home HD and peritoneal dialysis.
▪ Cross-training of healthcare personnel.
▪ Integration of dialysis services into emergency operation centers and disaster management plans.
▪ Routine disaster simulation exercises and preparedness audits.
▪ Investments in dialysis preparedness often generate broader benefits for hospitals and communities by strengthening infrastructure that can be utilized during other healthcare emergencies.

## Conclusion

Disaster management in HD centers is no longer optional—it is an ethical and operational necessity. The combination of dependency on utilities, complex logistics, and vulnerable patients demands integrated preparedness at every level. Through risk assessment, staff training, patient engagement, and coordinated policy frameworks, dialysis services can become resilient, ensuring that even amid chaos, the most fragile patients continue to receive the care they need. Particular attention must be directed toward strengthening disaster preparedness in LMICs, where fragile health systems, limited infrastructure, and resource constraints substantially magnify the risks faced by dialysis-dependent patients during emergencies.
